# Multilayer films for photon upconversion-driven photoswitching†

**DOI:** 10.1039/d4tc03513e

**Published:** 2024-11-26

**Authors:** Zhihang Wang, Beatrice E. Jones, Larissa G. Franca, Takashi Lawson, Martyn Jevric, Kasper Moth-Poulsen, Rachel C. Evans

**Affiliations:** a Department of Materials Science and Metallurgy, University of Cambridge 27 Charles Babbage Road Cambridge CB3 0FS UK rce26@cam.ac.uk; b Department of Chemistry and Chemical Engineering, Chalmers University of Technology Kemivagen 4 Gothenburg 412 96 Sweden; c Institute of Materials Science of Barcelona, ICMAB-CSIC, Bellaterra Barcelona 08193 Spain; d Catalan Institution for Research & Advanced Studies, ICREA Pg. Lluıs Companys 23 Barcelona Spain; e Department of Chemical Engineering, Universitat Politècnica de Catalunya, EEBE Eduard Maristany 10–14 08019 Barcelona Spain kasper.moth-poulsen@upc.edu

## Abstract

Photoswitchable materials are of significant interest for diverse applications from energy and data storage to additive manufacturing and soft robotics. However, the absorption profile is often a limiting factor for practical applications. This can be overcome using indirect excitation *via* complementary photophysical pathways, such as triplet sensitisation or photon upconversion. Here, we demonstrate the use of triplet–triplet annihilation upconversion (TTA-UC) to drive photoswitching of the energy storing photoswitch norbornadiene–quadricyclane (NBD–QC) in the solid-state. A photoswitchable bilayer polymer film, incorporating the TTA-UC sensitiser–emitter pair of platinum octaethylporphyrin (PtOEP) and 9,10-diphenylanthracene (DPA), was used to trigger the photoinduced [2+2] cycloaddition of NBD to form QC using visible instead of UV light. The isolated TTA-UC film showed green-to-blue upconversion, with a competitive upconversion efficiency of (1.9 ± 0.1%) for the solid-state in air. Direct photoswitching of the isolated NBD film was demonstrated with a narrow UV light source (340 nm). However, in the bilayer film, spectral overlap between the upconverted blue emission in the TTA-UC film and the absorbance band of the NBD film resulted in indirect photoswitching using visible green light (532 nm, 1 W cm^−2^), thus extending the spectral operational window of the photoswitching film. The results demonstrate proof-of-feasibility of TTA-UC-promoted photoswitching in the solid-state, paving the way for potential applications in light-harvesting devices and smart coatings, using a wider selection of irradiation wavelengths.

## Abstract 2

The use of triplet–triplet annihilation upconversion (TTA-UC) to drive photoswitching of the energy-storing norbornadiene–quadricyclane (NBD–QC) isomers in the solid-state is demonstrated.

## Graphical abstract

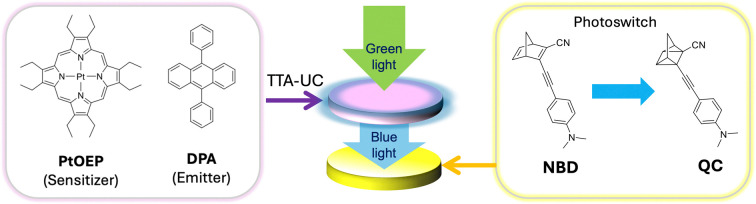

Triplet–triplet annihilation photon upconversion (TTA-UC) has emerged as a notable method for spectral conversion and photon control.^1^ First identified in 1962,^2,3^ TTA-UC has evolved substantially in recent years,^4^ finding diverse applications in photochemistry,^5^ photobiology,^6,7^ 3D printing,^8,9^ and solar conversion.^10–12^ As shown in Fig. 1, the mechanism of TTA-UC involves chromophore pairs (sensitiser and emitter) that combine two low-energy photons to form a single higher-energy photon. Given that the TTA-UC process relies on rapid energy transfer between the sensitiser and emitter, the physical properties of the host medium can also significantly influence the UC efficiency. This is measured by the upconversion quantum yield (UCQY), capped at a maximum of 50%.^13^ Recently, the combination of TTA-UC with organic photoswitchable molecules has gained attention as a promising approach to expand the spectral response of the systems.^10,12,14^ Photoswitches undergo photoisomerisation to a high-energy metastable state upon exposure to light and can revert to their original form through various triggering agents (*i.e.*, light,^15^ pH,^16^ heat,^17^ or catalyst^18^). Such materials are of interest for emerging technologies such as sensing and imaging,^19,20^ molecular electronics or logic gates,^21,22^ as well as molecular solar thermal energy storage (MOST).^17^

**Fig. 1:**
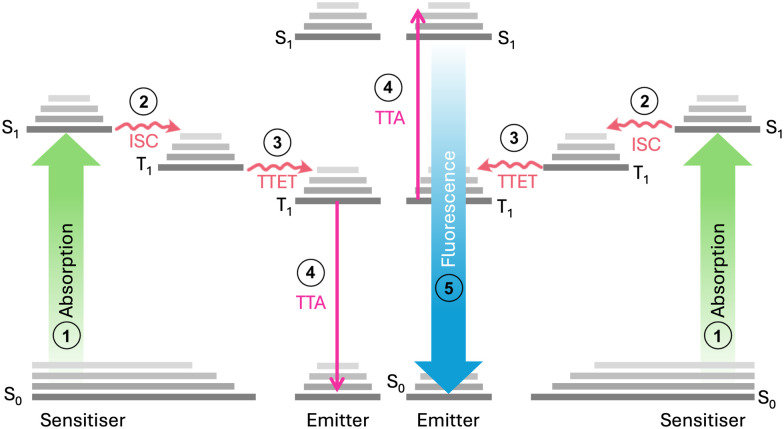
Triplet–triplet annihilation upconversion involves a multi-step photophysical interaction between two chromophores. The mechanism of TTA-UC involves: (1) photon absorption by the sensitiser PtOEP to create an excited singlet state (S_1_). (2) Relaxation to the excited triplet state (T_1_) *via* intersystem crossing (ISC). (3) Triplet sensitisers collide with emitters, producing triplet emitters through triplet–triplet energy transfer (TTET) if their energy levels overlap within the Dexter radius. (4) The triplet emitters then collide with each other, resulting in triplet–triplet annihilation (TTA). In this process, one molecule is excited to a higher energy level, while the other relaxes to the ground state. (5) The radiative relaxation of the higher energy state leads to photon emission *via* fluorescence.

In recent years, photoswitchable norbornadiene (NBD) chromophores have demonstrated considerable promise for MOST, due to their red-shifted absorption spectrum (NBD onset of absorption up to 529 nm),^23^ high photoisomerisation quantum yield (up to 97%),^24^ significant energy storage capacity (up to 559 kJ kg^−1^),^25^ and mechanical durability (up to 99% reversibility).^26^ NBDs can be converted to the corresponding quadricyclane (QC) photoisomer *via* a [2+2π] cycloaddition reaction under UV/blue irradiation. The back-conversion from QC to NBD can be triggered by heat or the use of a catalyst.^27,28^ Solid-state NBD systems could be used in various temperature regulation applications, including functional windows and thin films.^29^

While recent advances have shown that NBDs undergo efficient photoswitching upon direct excitation with UV light when incorporated within polymer films, this fails to exploit the visible region of the solar spectrum for energy harvesting and storage.^23^ One potential solution is to add an adjunct TTA-UC system, which absorbs light at wavelengths where the photoswitch is inactive and converts it to a higher energy where the photoswitch absorbs, thus expanding the useful spectral window.^5^ For instance, Albinson and coworkers selectively controlled both the ring-opening and ring-closing reactions of diarylethene photoswitches in toluene using a 532 nm laser light-triggered green-to-blue TTA-UC system.^30^ Börjesson *et al.* pioneered the use of a TTA-UC pair with a fulvalene diruthenium derivative, also in toluene, creating an artificial solar light-driven TTA-UC process, aiding in the charging of photoswitchable materials for MOST.^31^ Additionally, Castellano *et al.* used a Ru(ii) chromophore as a sensitiser and anthracene as an emitter to convert UV light into visible light, enabling anthracene to undergo dimerisation.^32^ However, in these studies, either the TTA-UC system or the photoswitchable molecules were in the solution state. The combination of both solid-state TTA-UC and NBD films could broaden the spectral operational range for photoswitching, thus mitigating challenges with optical penetration depth. To our surprise, such systems have, to the best of our knowledge, not yet been reported. Here, we report the design and performance of a bilayer film architecture that, for the first time, demonstrates TTA-UC promoted photoisomerisation of a photoswitchable molecule in the solid state. The system was designed as a bilayer film architecture for two reasons. Firstly, photoswitchable molecules can act as quenchers, introducing an additional non-radiative decay in the TTA process, which potentially reduces the number of upconverted photons produced.^33^ Secondly, most available TTA-UC-based films are susceptible to degradation under long-term or intense irradiation, and mixing the systems could introduce recycling challenges for the NBD molecules.^34^

In our bilayer film architecture, the TTA-UC layer comprises the well-established green-to-blue TTA-UC sensitiser–emitter pair, platinum octaethylporphyrin (PtOEP) and 9,10-diphenylanthracene (DPA), incorporated into a thin poly(vinyl acetate)polymer (PVAc) film (TTA-UC@PVAc film, Fig. 2a, c and Fig. S1, ESI†). PVAc was chosen because it is soluble in common organic solvents and exhibits excellent optical transparency, making it ideal for film manufacture and luminophore compatibility. To effectively use the upconverted emission from the TTA-UC@PVAc film, a NBD molecule whose absorption spectrum fully or partially overlaps with it should be selected for fabrication of the bilayer system. Here, a PVAc film containing a donor–acceptor substituted NBD, 2-cyano-3-((4-(dimethylamino)phenyl)ethynyl)norbornadiene, which exhibits spectral overlap with PtOEP and whose cyclability was previously demonstrated,^35,36^ was prepared to demonstrate proof-of-concept (NBD@PVAc, Fig. 2b, c and Fig. S2, ESI†). For both the TTA-UC@PVAc and NBD@PVAc films, the upper limit on film thickness is determined by light penetration depth, which will be dependent on the concentrations and molar absorption coefficients of the chromophores at the laser excitation wavelength and the wavelengths of the upconverted DPA emission, respectively. With this in mind, both films were prepared *via* drop casting, resulting in a net thickness of approximately 0.8 mm (Fig. 2d).

**Fig. 2:**
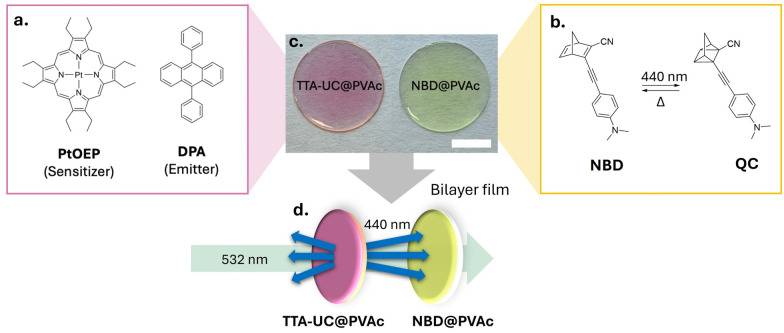
Design of an all-solid-state bilayer construct for TTA-UC-sensitised photoswitching. Chemical structures of (a) the sensitiser and emitter pair of TTA-UC and (b) the NBD photoswitch and QC photoisomer following [2+2] cycloaddition under UV/blue irradiation. (c) Transparent films are prepared by casting the chromophore(s) in a PVAc host (net thickness for both films is 0.78 ± 0.02 mm, scale bar: 0.5 cm). (d) In the bilayer system, a 532 nm laser triggers the TTA-UC process, producing 440 nm upconverted photons to induce the conversion of the NBD@PVAc film.

Fig. 3a shows the optical properties of the individual chromophores, photoswitches, and the PVAc host. While the Q-bands of the PtOEP sensitiser absorb in the green region (∼470–560 nm), the fluorescence emission of DPA exhibits spectral overlap with the absorption profile of NBD, suggesting that it should be an effective sensitiser for the photoswitch (spectral overlap: *J* = 2.3 × 10^3^ M^−1^cm^−1^, thus a spectral overlap of 0.1% with NBD in toluene, Section 2, ESI†).^35,37^ Importantly, the PVAc host also provides minimal contribution to the overall absorption, as supported by the transmittance spectrum, which is >95% across the spectral region of interest.

**Fig. 3:**
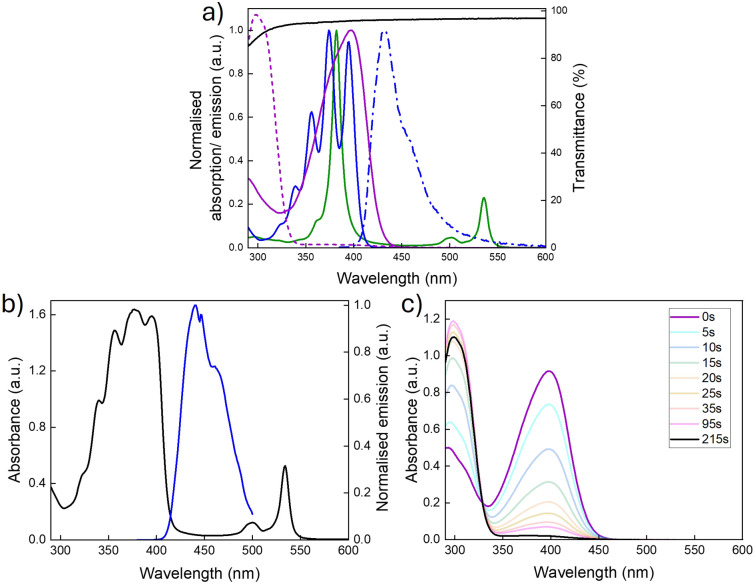
Optical properties of TTA-UC and photoswitch chromophores. (a) Absorbance spectrum of PtOEP (solid green line), DPA (solid blue line), NBD (solid purple line) and QC (dashed purple line), and fluorescence spectrum of DPA (blue dash-dotted line, *λ*_ex_ = 375 nm) in toluene (10^−2^ mM), and transmittance of a pure PVAc film (solid black line). (b) Absorbance and upconversion spectrum of TTA-UC@PVAc film under 532 nm laser excitation. (c) Absorbance spectrum showing the time-dependent photoswitching (0–215 seconds) of NBD to QC under a 340 nm LED light source.

The performance of the TTA-UC film was first investigated in isolation. The DPA : PtOEP ratio was tailored to determine the optimum concentration that maximised the TTA-UC efficiency while limiting aggregation in the PVAc host (Fig. S4, ESI†). An emitter:sensitiser ratio of 180 : 1 (27 mM to 0.15 mM, respectively) was shown to deliver the best performance and was used for all further studies. Fig. 3b presents the corresponding absorption spectra for the optimised TTA-UC@PVAc film and its upconversion emission spectrum. The absorbance clearly shows the distinct bands originating from the DPA (around 350–380 nm) and PtOEP (around 483 to 545 nm) ground-state (S_0_ → S_1_) transitions. Laser excitation at 532 nm selectively excites the PtOEP sensitiser, resulting in UC emission around 440 nm, which is characteristic of DPA.^38,39^ The threshold intensity, *I*_th_, for observing UC emission in the TTA-UC films was established to be 2 W cm^−2^ (Fig. S5, ESI†), much higher than other solid-state hosts (*e.g.* organic–inorganic hybrid ureasils^40^ – 27 mW cm^−2^), with a UC quantum yield of 1.9% ± 0.1% under ambient conditions (*i.e.* no oxygen removed).

The UC and phosphorescence decay curves (in air) are both best described by a biexponential decay function (see Sections 5 and 6, ESI†), with average lifetimes 〈*τ*_UC_〉 = 8.5 ms (at 440 nm, Fig. S6 and Table S1, ESI†) and 〈*τ*_phos_〉 = 89.8 μs (at 665 nm, Fig. S7 and Table S2, ESI†), respectively. Attribution of physical meaning to specific lifetime components is challenging, as several factors may contribute including oxygen quenching and a distribution of chromophore sites within the host. The phosphorescence of a control film in air, containing PtOEP only, also decayed biexponentially, with a short-lived lifetime, *τ*_1_ ∼23 μs, assigned to O_2_-quenched sites and a longer lifetime component, *τ*_2_ ∼ 61 μs, assigned to the natural phosphorescence lifetime (Fig. S7 and Table S2, ESI†), which is consistent with literature reports for PtOEP in polymer films in air.^41^ Interestingly, the corresponding lifetimes for the TTA-UC@PVAc film in air are longer lived (*τ*_1_ ∼ 37.7 μs, *τ*_2_ ∼ 100 μs) and do not exhibit monoexponential decay even in an N_2_ atmosphere (*τ*_1_ ∼ 60.5 μs, *τ*_2_ ∼ 110 μs), see Table S2 (ESI†), indicating that the biexponential behaviour is primarily a function of the film environment (*e.g.* availability of different chromophore sites), rather than O_2_ quenching. Moreover, the longer 〈*τ*_phos_〉 for the TTA-UC@PVAc film reveals that the addition of DPA reduces the contribution of the quenched species (A_1_ ∼ 34.3%), which now compete for interaction with the emitter molecules. This results in a higher contribution of *τ*_2_, assigned to PtOEP triplet states in sites without accessible DPA neighbours, leading to the increased 〈τ_phos_〉 in the TTA-UC@PVAc film.

The optical and kinetic properties of the NBD@PVAc photoswitchable film were examined next. In toluene solution, the absorption onset was at 456 nm, with a peak maximum at 398 nm (Fig. 3a). Upon integration into the PVAc film, the peak absorption of NBD remained essentially unchanged (*λ*_max_ = 397 nm, Fig. 3c). The back-conversion kinetics for QC to NBD in the PVAc matrix were shown to have a half-life, *t*_1/2_ of 3.8 hours (226 minutes) at 25 °C, following the Eyring plot (Fig. S8, ESI†). This result is slightly shorter than the reported half-life for this photoswitch in toluene of 5.05 hours at room temperature.^35^ We note that the photoisomerisation quantum yield for NBD to QC in the film may be slightly lower than in solution, since NBD molecules tend to photoisomerise more readily when irradiated in solution compared to the solid state. However, such deviations are challenging to quantify accurately, as standard chemical actinometry methods used to measure the photoisomerisation quantum yield can lead to significant errors, particularly for films of such small size.^24^

Lastly, the photoswitchable and TTA-UC films were investigated in a bilayer architecture, with the two films physically attached to each other (Fig. S4, ESI†), to evaluate the potential for sensitisation of the NBD to QC photoconversion using the TTA-UC@PVAc film. Since the absorbance spectrum of the NBD absorption overlaps well with the UC emission spectrum of the TTA-UC system, wavelengths between 400 nm and 450 nm emitted by the DPA emitter should induce efficient NBD conversion in the photoswitchable film to the QC state following the sensitisation mechanism proposed for solution-state analogues (Section 8, ESI†).^31^ To illustrate the TTA-UC-assisted conversion in the photoswitchable film, a 532 nm laser at 1 W cm^−2^ was used to excite the sensitisers within the TTA-UC film (Fig. 4a). A control experiment was conducted to demonstrate that the laser alone would not trigger the photoconversion of NBD by replacing the slide with the TTA-UC film with an empty glass substrate. By tracking the absorbance at 397 nm, no significant change in the absorption was observed for the sample without the TTA-UC film (1 W cm^−2^ of a 532 nm laser, Fig. 4b). However, the presence of the TTA-UC film led to a significant increase in conversion rate upon irradiation (nearly 100% conversion after irradiating for 2 min). Although the emission signal of the TTA-UC@PVAc film was observed to decrease over time (Section 9, ESI†), a common issue associated with the photobleaching of organic dyes under prolonged light exposure, this clearly demonstrates that the TTA-UC film is able to drive the photoisomerisation of NBD, thus expanding the spectral operation window.

**Fig. 4:**
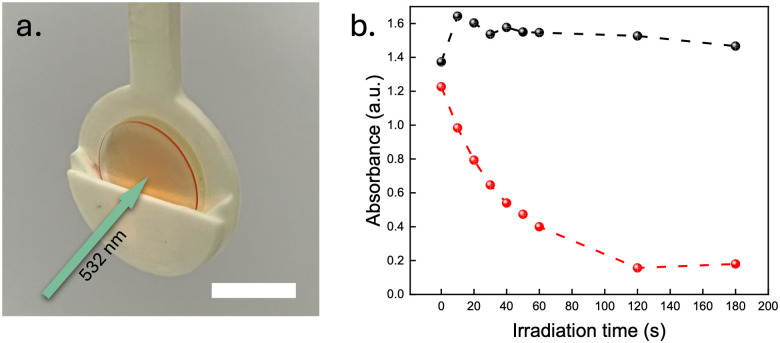
TTA-UC sensitised photoconversion of NBD to QC in a solid-state PVAc film in air. (a) Photograph of the TTA-UC@PVAc/NBD@PVAc bilayer film architecture in the sample holder. The green arrow indicates the direction of the 532 nm laser beam (scale bar: 0.5 cm). (b) Photoconversion of the NBD@PVAc film to QC@PVAc as monitored by the decrease in NBD absorbance at *λ*_max_ = 397 nm under direct irradiation (532 nm, black circles) and through sensitisation the TTA-UC@PVAc film (red circles).

## Conclusions

In summary, this study showcased the effective use of TTA-UC to drive the photoconversion of NBD to QC in a bilayer film construct. To the best of our knowledge, this is the first demonstration of a fully integrated, all-solid-state photoswitchable film that can be indirectly sensitised by TTA-UC. The advantages of this approach include solvent elimination and a move away from UV irradiation to visible frequencies, which should extend both the range and lifetime of any practical applications. The fabrication of highly transparent PVAc host films is straightforward, and the bilayer structure can be created by simply attaching two films, thereby avoiding material mixing. The TTA-UC film demonstrated an upconversion efficiency of 1.9 ± 0.1%. Direct photoswitching of the NBD film using UV excitation proceeded with a comparable *t*_1/2_ to that in solution, suggesting the kinetics of thermal backconversion are not impacted by inclusion in the PVAc host. Finally, the combination of the two films as a bilayer system demonstrated highly efficient conversion of NBD to QC *via* indirect excitation using green light, illustrating that TTA-UC can be used to expand the spectral window for the conversion of photoswitches in the solid state. With future leveraging of the latest developments in heavy metal-free sensitisers,^42,43^ efficient solar-driven TTA-UC pairs,^44^ and more finely tuned photoswitches designed in recent years, this work paves the way for investigating TTA-UC pairs that exhibit near-infrared (NIR)-to-Vis^12,45,46^ or Vis-to-UV^42,44^ conversions. Technologies like MOST systems^17^ and hybrid photovoltaic cells^47^ could particularly benefit from TTA-UC enhanced solar energy conversion and collection *via* photoswitching, as the flexibility and ease of use of such solid films could significantly enhance their deployment and applicability.

## Data availability

Data for this article are available at the University of Cambridge Apollo repository at https://doi.org/10.17863/CAM.113061.

## Conflicts of interest

There are no conflicts to declare.

## Supplementary Material

TC-012-D4TC03513E-s001

## References

[cit1] Zhao J., Wu W., Sun J., Guo S. (2013). Chem. Soc. Rev..

[cit2] Parker C., Hatchard C. (1962). Proc. R. Soc. Lond. A.

[cit3] Parker C. A., Hatchard C. G. (1962). Proc. Chem. Soc..

[cit4] Singh-Rachford T. N., Castellano F. N. (2010). Coord. Chem. Rev..

[cit5] Huang L., Han G. (2024). Nat. Rev. Chem..

[cit6] Lin W., Li J., Feng H., Qi F., Huang L. (2023). J. Anal. Test..

[cit7] Askes S. H. C., Kloz M., Bruylants G., Kennis J. T. M., Bonnet S. (2015). Phys. Chem. Chem. Phys..

[cit8] O’Dea C. J., Isokuortti J., Comer E. E., Roberts S. T., Page Z. A. (2024). ACS Cent. Sci..

[cit9] Sanders S. N., Schloemer T. H., Gangishetty M. K., Anderson D., Seitz M., Gallegos A. O., Stokes R. C., Congreve D. N. (2020). Nature.

[cit10] Naimovičius L., Bharmoria P., Moth-Poulsen K. (2023). Mater. Chem. Front..

[cit11] Gray V., Dzebo D., Abrahamsson M., Albinsson B., Moth-Poulsen K. (2014). Phys. Chem. Chem. Phys..

[cit12] Bharmoria P., Bildirir H., Moth-Poulsen K. (2020). Chem. Soc. Rev..

[cit13] Zhou Y., Castellano F. N., Schmidt T. W., Hanson K. (2020). ACS Energy Lett..

[cit14] Kuntze K., Isokuortti J., van der Waals J. J., Laaksonen T., Crespi S., Durandin N. A., Priimagi A. (2024). Chem. Sci..

[cit15] Ji Y., Yu H. (2024). J. Mater. Chem. C.

[cit16] Dreos A., Wang Z., Tebikachew B. E., Moth-Poulsen K., Andréasson J. (2018). J. Phys. Chem. Lett..

[cit17] Wang Z., Erhart P., Li T., Zhang Z.-Y., Sampedro D., Hu Z., Wegner H. A., Brummel O., Libuda J., Nielsen M. B., Moth-Poulsen K. (2021). Joule.

[cit18] Wang Z., Roffey A., Losantos R., Lennartson A., Jevric M., Petersen A. U., Quant M., Dreos A., Wen X., Sampedro D., Börjesson K., Moth-Poulsen K. (2019). Energy Environ. Sci..

[cit19] Abdollahi A., Roghani-Mamaqani H., Razavi B. (2019). Prog. Polym. Sci..

[cit20] Minoshima M., Kikuchi K. (2017). JBIC, J. Biol. Inorg. Chem..

[cit21] Huang X., Li T. (2020). J. Mater. Chem. C.

[cit22] Karar M., Shit P., Halder B., Mallick A., Majumdar T. (2018). ChemistrySelect.

[cit23] Petersen A. U., Hofmann A. I., Fillols M., Mansø M., Jevric M., Wang Z., Sumby C. J., Müller C., Moth-Poulsen K. (2019). Adv. Sci..

[cit24] Kjaersgaard A., Hölzel H., Moth-Poulsen K., Nielsen M. B. (2022). J. Phys. Chem. A.

[cit25] Mansø M., Petersen A. U., Wang Z., Erhart P., Nielsen M. B., Moth-Poulsen K. (2018). Nat. Commun..

[cit26] Waidhas F., Jevric M., Fromm L., Bertram M., Görling A., Moth-Poulsen K., Brummel O., Libuda J. (2019). Nano Energy.

[cit27] Orrego-Hernández J., Dreos A., Moth-Poulsen K. (2020). Acc. Chem. Res..

[cit28] Yoshida Z.-i (1985). J. Photochem..

[cit29] Refaa Z., Hofmann A., Castro M. F., Hernandez J. O., Wang Z., Hölzel H., Andreasen J. W., Moth-Poulsen K., Kalagasidis A. S. (2022). Appl. Energy.

[cit30] Larsson W., Morimoto M., Irie M., Andréasson J., Albinsson B. (2023). Chem. – Eur. J..

[cit31] Börjesson K., Dzebo D., Albinsson B., Moth-Poulsen K. (2013). J. Mater. Chem. A.

[cit32] Islangulov R. R., Castellano F. N. (2006). Angew. Chem., Int. Ed..

[cit33] Cristol S. J., Kaufman R. L. (1980). J. Photochem..

[cit34] Spikes J. D. (1992). Photochem. Photobiol..

[cit35] Quant M., Lennartson A., Dreos A., Kuisma M., Erhart P., Börjesson K., Moth-Poulsen K. (2016). Chem. – Eur. J..

[cit36] Dreos A., Börjesson K., Wang Z., Roffey A., Norwood Z., Kushnir D., Moth-Poulsen K. (2017). Energy Environ. Sci..

[cit37] Gray V., Moth-Poulsen K., Albinsson B., Abrahamsson M. (2018). Coord. Chem. Rev..

[cit38] Yanai N., Kimizuka N. (2020). Angew. Chem., Int. Ed..

[cit39] Bennison M. J., Collins A. R., Zhang B., Evans R. C. (2021). Macromolecules.

[cit40] Collins A. R., Zhang B., Bennison M. J., Evans R. C. (2024). J. Mater. Chem. C.

[cit41] Douglas P., Eaton K. (2002). Sens. Actuators, B.

[cit42] Uji M., Harada N., Kimizuka N., Saigo M., Miyata K., Onda K., Yanai N. (2022). J. Mater. Chem. C.

[cit43] Watanabe S., Mizukami K., Kimizuka N., Yasuda T. (2024). J. Mater. Chem. C.

[cit44] Harada N., Sasaki Y., Hosoyamada M., Kimizuka N., Yanai N. (2021). Angew. Chem., Int. Ed..

[cit45] Bharmoria P., Ghasemi S., Edhborg F., Losantos R., Wang Z., Mårtensson A., Morikawa M.-A., Kimizuka N., Dumoulin F., Albinsson B., Moth-Poulsen K. (2022). Chem. Sci..

[cit46] Sasaki Y., Amemori S., Kouno H., Yanai N., Kimizuka N. (2017). J. Mater. Chem. C.

[cit47] Wang Z., Hölzel H., Fernandez L., Aslam A. S., Baronas P., Orrego-Hernández J., Ghasemi S., Campoy-Quiles M., Moth-Poulsen K. (2024). Joule.

