# Supplementary material for: Multilayer films for photon upconversion-driven photoswitching
Source: J Mater Chem C Mater. 2024 Nov 26;12(47):19030–4. doi: 10.1039/d4tc03513e (PMC11589804; doi:10.1039/d4tc03513e)
Supplement: TC-012-D4TC03513E-s001 [file TC-012-D4TC03513E-s001.pdf]

Supplementary Information (SI) for Journal of Materials Chemistry C.
This journal is © The Royal Society of Chemistry 2024

# Supporting Information

# Multilayer Films for Photon Upconversion-Driven Photoswitching

*Zhihang Wang,*[a] *Beatrice E. Jones,*[a] *Larissa G. Franca,*[a] *Takashi Lawson,*[a] *Martyn Jevric,*[b] *Kasper Moth-Poulsen*[b,c,d,e]* *and Rachel C. Evans*[a]*

[a] Department of Materials Science & Metallurgy, University of Cambridge, 27 Charles Babbage Road, CB3 0FS, U.K.

[b] Department of Chemistry and Chemical Engineering, Chalmers University of Technology, Kemivagen 4, Gothenburg 412 96, Sweden.

[c] Institute of Materials Science of Barcelona, ICMAB-CSIC, Bellaterra, Barcelona, 08193, Spain.

[d] Catalan Institution for Research & Advanced Studies, ICREA, Pg. Lluís Companys 23, Barcelona, Spain.

[e] Department of Chemical Engineering, Universitat Politècnica de Catalunya, EEBE, Eduard Maristany 10–14, 08019 Barcelona, Spain.

* Corresponding Author: Prof. Rachel C. Evans (rce26@cam.ac.uk); Prof. Kasper Moth-Poulson (kasper.moth-poulsen@upc.edu)

# Table of Contents

1 Experimental Methods ........ 3

1.1 Materials ........ 3

1.2 Fabrication of TTA-UC@PVAc films ........ 3

1.3 Fabrication of NBD@PVAc films ........ 3

1.4 Bilayer architecture ........ 4

1.5 Optical characterisation ........ 4

1.6 Photoisomerisation kinetics ........ 7

2 Calculation of spectral overlap integral ........ 8

3 Optimisation of the sensitiser:emitter ratio in TTA-UC@PVAc films ........ 8

4 Threshold intensity study on TTA-UC@PVAc film ........ 9

5 UC lifetime studies ........ 9

6 Phosphorescence lifetime measurements ........ 10

7 Kinetic studies on converted NBD@PVAc (QC@PVAc) film ........ 12

8 TTA-UC assisted photoswitching mechanism ........ 12

9 TTA-UC@PVAc film fatigue test ........ 13

10 References ........ 13

# 1 Experimental Methods

## 1.1 Materials

Poly(vinyl acetate) (~M.W. 170,000) was purchased from Fisher Scientific. Platinum octaethylporphyrin (PtOEP) and toluene (ACS reagent, ≥99.5%) were purchased from Sigma Aldrich. 9,10-Diphenylanthracene was purchased from Alfa Aesar. 2-Cyano-3-((4(dimethylamino)phenyl)ethynyl)norbornadiene (NBD) was synthesised following a method reported previously. [1]

## 1.2 Fabrication of TTA-UC@PVAc films

Figure S1 shows a schematic representation of process used to fabricate TTA-UC-PVAc films. A solution containing 1 mL of 0.15 mM PtOEP in toluene and 1 mL of 27 mM DPA in toluene was added to 400 mg of PVAc polymer. Subsequently, 150 µL of the resultant TTA-UC@PVAc in toluene solution was drop-cast onto a glass slide with a diameter of 12 mm and a thickness of 0.16 mm. The film was then dried in a 70°C oven for 2 hours to prepare it for further characterisation.

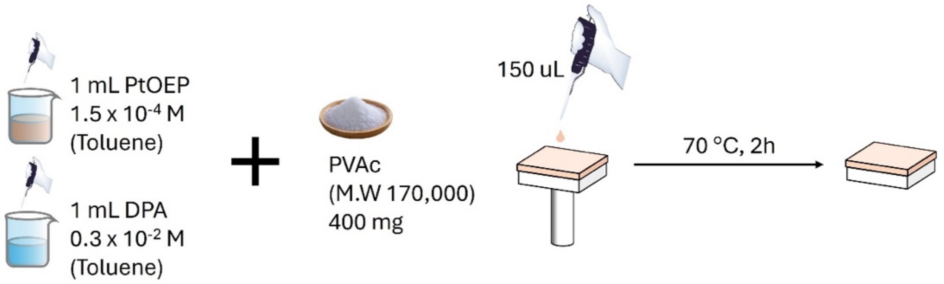


**Figure S1.** Schematic representation of the TTA-UC@PVAc film fabrication process.

## 1.3 Fabrication of NBD@PVAc films

Figure S2 shows a schematic representation of process used to fabricate NBD@PVAc films. 1 mL NBD in toluene solution (0.15 mM) was mixed with 400 mg PVAc polymer. 150 µL of the sticky mixed solution was drop casted on a glass substrate, then put in a 70 °C for 2 h before characterisation.

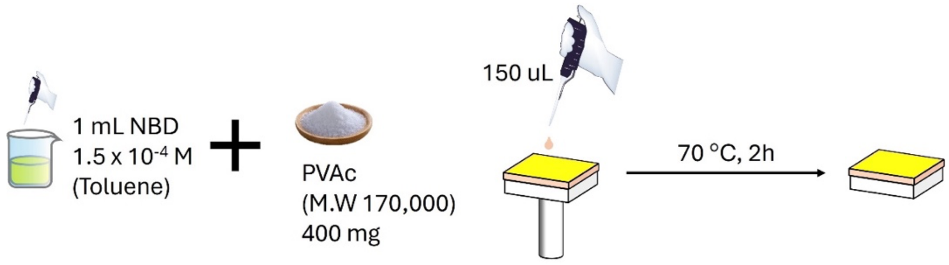


**Figure S2.** Schematic representation of the NBD@PVAc film fabrication process.

## 1.4 Bilayer architecture

Figure S3 shows the fabrication of the bilayer architecture, where the TTA-UC@PVAc and NBD@PVAc films on glass substrates were physically attached to each other face-to-face.

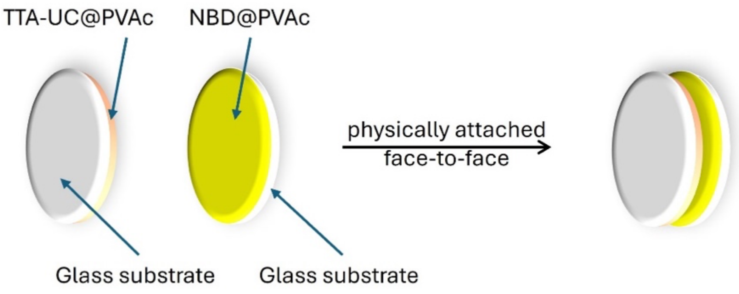


**Figure S3.** Schematic representation of the bilayer architecture system.

## 1.5 Optical characterisation

UV-Vis absorption/transmittance spectra were measured with a Cary-60 spectrophotometer using wavelength scan from 290-800 nm with a resolution of 1 nm at a scan speed of 600 nm $min^{-1}$ and an average time of 0.1 s. Solid-state samples (with glass substrate) were directly mounted to the sample holder. Conversion spectra of NBD@PVAc film with and without TTA-UC@PVAc film were collected using an Ocean Optics Flame spectrometer (integration time 100 ms), together with an Ocean Optics DH-2000 white light source.

The upconversion (UC) emission spectra, phosphorescence spectra, UC quantum yield (UCQY) and UC fluorescence lifetime and phosphorescence lifetimes of all samples were measured with an FLS 1000 TCSPC spectrometer (Edinburgh Instruments Ltd.). Samples were excited with a 532 nm laser (MGL-III-532, 200 mW), with a short-pass filter (cut-off 500 nm, Thorlabs) applied in front of the detector for upconversion measurements. The laser power was adjusted using Thorlabs PM100A Power Meter Console combined with a S120VC Si photodiode power sensor (range: 200-1100 nm). Unless otherwise stated all measurements were performed in air at room temperature. For measurements under $N_2$ atmosphere, $N_2$ was purged for 2.5 hours.

The threshold intensity, $I_{th}$, of the TTA-UC@PVAc film samples were measured with an FLS 1000 TCSPC spectrometer (Edinburgh Instruments Ltd.). Samples were excited with a 532 nm laser, with a short-pass filter (cut-off 500 nm, Thorlabs) applied in front of the detector for upconversion measurements. The power was chosen between 9 mW $cm^{-2}$ to 9091 mW $cm^{-2}$.

The UCQY was measured with an integrating sphere (SNS125 5-inch sphere, three windows, International Light Technologies). The sample was loaded at the center of the sphere by a sample holder. A baffle is placed in front of the observation window, which blocks any scattering and reflection of the

laser from the sample surface. The angle of the sample holder is adjustable. The normal direction of the sample holder is 22.5° to the excitation beam line, which leads the reflection of the laser to the inner surface of the sphere.

The laser power was measured with a photodiode before each UCQY measurement. Both the emission of the sample (380-500 nm) and scattering of the laser beam (530-534 nm) were measured. A neutral density filter (O.D.=3.0) was placed before the excitation beam for the scattering intensity measurements. Six data sets were collected to calculate the UCQY of each sample: 1. sample in the path of the beam – "in fluorescence"; 2. sample in scattering; 3. sample facing away from beam – "out of fluorescence", 4. sample out of scattering; 5. empty sphere fluorescence; 6. empty sphere scattering.

Three sets of data were obtained for three identical samples. The parallel data sets were calculated separately, which gives three UCQY results for each sample, and the reported UCQY is the average of these data, along with the standard deviation of the measurements. During the 'sample in beam' measurement, the sample was facing toward both the excitation window and the observation window, while in the sample-out mode, the holder was turned 180° to have the back of the holder facing the windows. For the fluorescence measurement (Data sets 1 and 3), the bandwidth was 1 nm for the detector, and the scan step was 1 nm per data point with duration of 1 second, scanned from 380 nm to 500 nm. For the scattering measurement (Data sets 2 and 4), the bandwidth was 1 nm for the detector, and the scan step was 0.1 nm per data point with duration of 0.1 second, scanned from 530 nm to 534 nm. The transmittance of the filter at the excitation wavelength was measured with a UV-Vis absorption spectrometer (DS5, Edinburgh Instruments Ltd.), taking the average over 10 parallel measurements. The empty-sphere data sets (Data sets 5 and 6) were collected at the beginning of the measurement, under the same conditions of the sample-in measurement, which were shared in all calculations of samples measured in the same day. During the calculation, all data were corrected by the transmittance of each filter used, and normalised based on the slit-width, scan step and the scan duration used.

The UCQY was calculated using the experimental approach described by Porrès *et al.* [2] and the following formulae: [3, 4]

$$\Phi_{\mathrm{UC}} = \frac{E_{x,in} - (1-A)E_{x,out}}{AL_{b,in}} \qquad \text{Eq. S1}$$

where $A$ is the percentage of the photons absorbed directly by the sample, which is corrected by removing the secondary absorption from the sphere-reflected photons:

$$A = \frac{L_{x,out} - L_{x,in}}{L_{x,out}} \qquad \text{Eq. S2}$$

where $E$ is the integrated photon counts from emission spectra, and $L$ is the integrated photon counts from the scattering spectra. $x$ delineates sample, while $b$ is blank. *In* means the sample was in the path of the excitation beam, and *out* delineates the sample is out of the beam line. A quantum yield is defined as the ratio of absorbed to emitted photons, meaning the UCQY is limited to 50% since this is a bimolecular process. While some papers report this as a normalised value, UCQY is reported to its un-normalised value here, to a maximum of 50%.

Emission decay (UC fluorescence or phosphorescence) measurements were performed using the multi-channel scaling (MCS) method on the FLS1000 PL spectrometer. The emission decay was recorded using a highspeed photomultiplier tube (PMT-980) equipped with TCC2 counting electronics. The UC fluorescence decay profile was measured using 532 nm laser excitation (MGL-III-532 laser), with a short-pass filter (cut-off 500 nm, Thorlabs) applied in front of the detector. The pulse repetition rate was 12.5 kHz (80 μs), the laser power was set to its maximum, and the pulse width was gradually increased until more than 1000 counts/s was observed at 440 nm. For phosphorescence lifetimes at 665 nm, a long-pass 590 nm filter was used in place of the 500 nm cut off filter and the repetition rate increased to 1 kHz (1 ms) for TTA-UC film, and 500 Hz (2 ms) for PtOEP only film. The instrument response function (IRF) was measured using with a $SiO_2$ particle suspension solution (Ludox® colloidal silica) using a neutral density filter (OD=3) in front of the excitation source, and with no emission filter.

Individual tail-fits were applied to each emission decay curve using the FAST software package (Edinburgh Instruments) using a multiexponential decay function:

$$I(t) = \sum_{i}^{n} \alpha_i e^{-t/\tau_i} \qquad \text{Eq. S3}$$

where $\alpha_i$ and $\tau_i$ are the amplitude and lifetime of the $i^{th}$ component, respectively, and $\alpha_i$ is normalized to unity for n components. The goodness of fit was assessed using the reduced chi-square statistics, $\chi^2$, and the randomness of the residuals. [5] To facilitate comparison of the emission decay behaviour between different samples, the average lifetime and fractional contributions ($f_i$) are also reported, which for a bi-exponential decay is given by:

$$\langle\tau\rangle = \frac{\alpha_1\tau_1^2 + \alpha_2\tau_2^2}{\alpha_1\tau_1 + \alpha_2\tau_2} = f_1\tau_1 + f_2\tau_2 \qquad \text{Eq. S4}$$

where:

$$f_i = \frac{\alpha_1\tau_1}{\sum_j^n \alpha_j\tau_j} \qquad \text{Eq. S5}$$

## 1.6 Photoisomerisation kinetics

UV-Vis absorbance spectra of the photoisomerisation kinetics were measured using a Perkin Elmer Lambda 750 spectrometer with a slit width of 1 nm and a scan speed of 266.75 nm min$^{-1}$. Measurements were taken at 1 nm intervals from 500 – 290 nm. Temperature control was achieved using Perkin Elmer Peltier temperature controller 201 with water cooling. Solid film samples were sandwiched between two quartz slides, and a background of the empty slides subtracted. The UV-Vis absorbance spectrum in the native state was first taken at 25 °C. The sample was irradiated for 10 minutes under UV light (Thorlabs 340 nm, minimum 53 mW Mounted LED, 700 mA). Spectra were then taken every 2 minutes for 52 measurements at temperatures of 25, 35 and 45 °C.

The thermal back-conversion of the photoisomer to the parent molecule can be described by a pseudo-first-order kinetic equation:

$$[A(\lambda)](t) = [A_0(\lambda)]e^{-kt} \qquad \text{Eq. S6}$$

where $[A(\lambda)](t)$, $[A_0(\lambda)]$ are the actual and initial concentrations, respectively; $k$ is the reaction rate constant at a specific temperature in s$^{-1}$ and $t$ is the reaction time in s.

The reaction rate constant depends on the thermal activation barrier of the back conversion. This energy barrier can be quantitatively described using the Eyring equation.

$$k = \frac{k_B T}{h} e^{-\frac{\Delta G^{\ddagger}}{RT}} \qquad \text{Eq. S7}$$

where $k_B$ is Boltzmann's constant in J K$^{-1}$, $h$ is Planck's constant in J s, and $\Delta G^{\ddagger}$ refers to the Gibbs free energy in J mol$^{-1}$. In addition, the Gibbs energy can be written in relation to enthalpy and entropy:

$$\Delta G^{\ddagger} = \Delta H^{\ddagger}_{therm} - T\Delta S^{\ddagger} \qquad \text{Eq. S8}$$

where $\Delta H^{\ddagger}_{therm}$ represents the activation enthalpy of the thermal back-conversion and $\Delta S^{\ddagger}$ is the entropy. Consequently, equation S8 can also be expressed as:

$$ln\frac{k}{T} = ln\frac{k_B}{h} - \frac{\Delta H^{\ddagger}_{therm}}{RT} + \frac{\Delta S^{\ddagger}}{R} \qquad \text{Eq. S9}$$

At a specific temperature, $ln\ k/T$ is a linear equation dependent on $1/T$, and, therefore, $k$ at any given temperature can be determined

The half-life of the photoisomer at a specific temperature corresponds to a moment where $[A(\lambda)]$ equals $[A_0(\lambda)]/2$. From equation S6, it can then be written as:

$$t_{1/2} = \frac{\ln(2)}{k} \quad \text{Eq. S10}$$

## 2 Calculation of spectral overlap integral

The spectral overlap between an emitting molecule and an absorbing molecule can be quantified by calculating the overlap integral $J(\lambda)$. The upconversion emission spectrum ($\varphi_{em}(\lambda)$) was normalized to unity, so that the integral over all wavelengths equals one. The molar absorption coefficient of NBD ($\varepsilon_{abs}(\lambda)$) was taken from literature[1] and used to plot the absorption spectrum. The $J(\lambda)$ was then obtained from:

$$J(\lambda) = \int \varphi_{em}(\lambda) \cdot \varepsilon_{abs}(\lambda) \cdot d\lambda \quad \text{Eq. S11}$$

The spectral overlap percentage with NBD in toluene is calculated as:

$$\text{Spectral overlap percentage (\%)} = \frac{J(\lambda)}{\int \varepsilon_{abs}(\lambda) \cdot d\lambda} \quad \text{Eq. S12}$$

## 3 Optimisation of the sensitiser:emitter ratio in TTA-UC@PVAc films

To test the optimal DPA and PtOEP mixing ratio to maximise TTA-UC and minimise aggregation of the chromophores in the solid state, films with different DPA:PtOEP molar ratios were prepared and the upconverted emission intensity at 440 nm was monitored under identical measurement conditions. It was observed that a DPA:PtOEP molar ratio exceeding 180:1 could lead to aggregation of DPA (Figure S4).

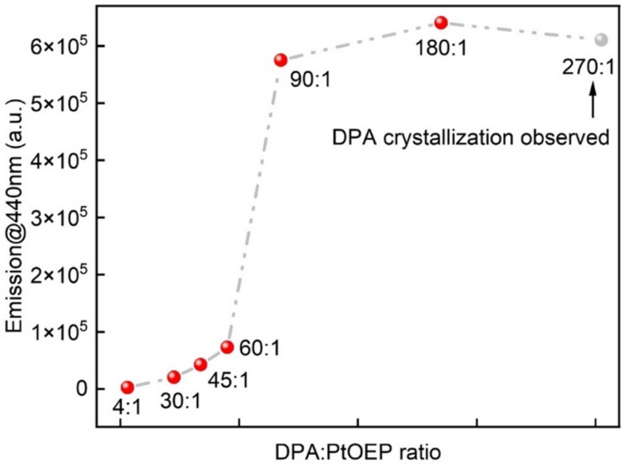


**Figure S4**. Optimisation of the DPA and PtOEP mixing ratio PVAc films by monitoring the upconversion emission intensity at 440 nm. It was observed that a DPA:PtOEP molar ratio exceeding 180:1 could lead to aggregation of DPA.

## 4 Threshold intensity study on TTA-UC@PVAc film

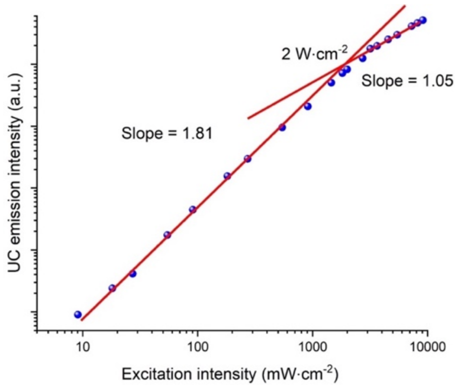


**Figure S5.** Log–log plot of the excitation intensity dependence of UC emission intensity for the TTA-UC@PVAc film. The solid lines correspond to linear fits of slopes 1.81 and 1.05 in the low and high excitation intensity regimes.

## 5 UC lifetime studies

Figure S6 shows the UC emission decay curves and corresponding fits for the TTA-UC@PVAc film in air or $N_2$ atmosphere. All decay curves required tail-fitting to a bi-exponential function, with the quality of fit evaluated by the evenness of the residuals. We selected the DPA (27 mM) and PtOEP (0.15 mM) as the emitter/sensitiser concentration to be used in all samples.

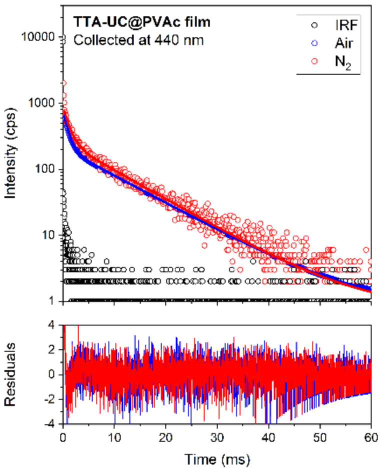


**Figure S6.** Upconversion decay curves (open symbols), fits (solid lines) and residuals of TTA-UC@PVAC film in air or $N_2$ atmosphere, obtained from excitation at 532 nm and detection at 440 nm (with a 550 nm short-pass filter). All fits were obtained via tail-fitting to a bi-exponential function.

**Table S1.** Summary of fitting parameters obtained for double exponential tail fits to the upconversion lifetime decay curves (Figure S5) of TTA-UC@PVAc film measured in air or $N_2$ atmosphere. $\lambda_{ex}$ = 532 nm, $\lambda_{em}$ = 440 nm.

| **TTA-UC@PVAc film** | $\chi^{2\,a}$ | **Component** | $\alpha_i$ $^b$ **(%)** | $f_i$ **(%)**$^c$ | $\tau_i$ **(ms)**$^d$ | **<$\tau_i$> (ms)** |
|---|---|---|---|---|---|---|
| In Air | 1.194 | 1 | 68.3 | 16.4 | 0.9 | 8.5 |
| | | 2 | 31.7 | 83.6 | 10.0 | |
| In $N_2$ | 1.150 | 1 | 66.2 | 18.2 | 1.1 | 8.2 |
| | | 2 | 33.8 | 81.8 | 9.8 | |

$^a$ $\chi$-squared, a measure of goodness of fit. $^b$ Normalised pre-exponential factor for $i^{th}$ component. $^c$ Fractional contribution of the $i^{th}$ component. $^d$ Lifetime of the $i^{th}$ component.

# 6 Phosphorescence lifetime measurements

Figure S7 shows the phosphorescence decay curves and corresponding fits for PtOEP in PVAc films, in the presence (in air or $N_2$ atmosphere) and absence of the DPA emitter (in air). All decay curves required a tail-fitting to a bi-exponential function, with the quality of fit evaluated by the evenness of the residuals.

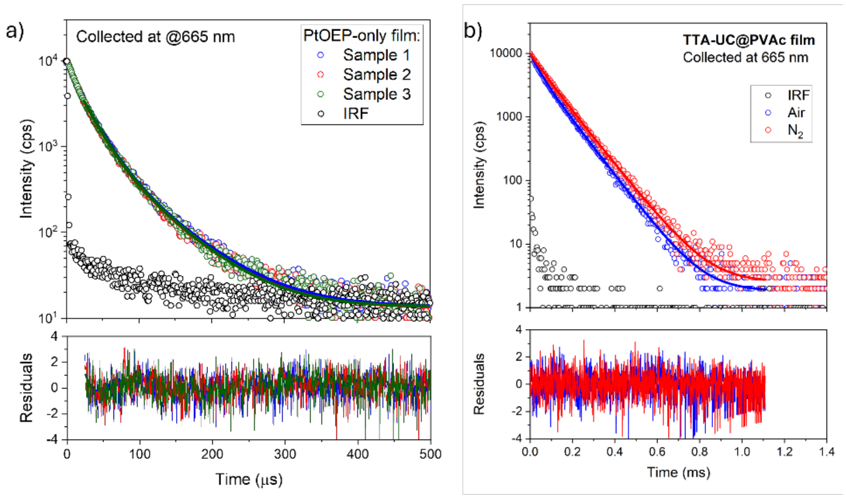


**Figure S7.** Phosphorescence decay curves (open symbols), fits (solid lines) and residuals of (a) PtOEP only film in air and (b) TTA-UC@PVAc film in air or $N_2$ atmosphere, obtained from excitation at 532 nm and detection at 665 nm (with a long-pass 590 nm filter). All fits were obtained via tail-fitting to a bi-exponential function.

**Table S2.** Summary of fitting parameters obtained for double exponential tail fits to the upconversion lifetime decay curves (Figure S6) of PtOEP only film in air and TTA-UC@PVAc film in air or $N_2$ atmosphere. $\lambda_{ex}$ = 532 nm, $\lambda_{em}$ = 665 nm.

| | | $\chi^{2\,a}$ | **Component** | $\alpha_i$ **(%)**[b] | $f_i$ **(%)**[c] | $\tau_i$ **(µs)**[d] | **<$\tau_i$> (µs)** | **<$\tau_i$>$_{mean}$ (µs)** |
|---|---|---|---|---|---|---|---|---|
| PtOEP only film<br><br>In Air | Sample1 | 1.157 | 1 | 76.4 | 54.8 | 23.9 | 42.0 | 41.3±0.4 µs |
| | | | 2 | 23.6 | 45.2 | 63.9 | | |
| | Sample2 | 1.140 | 1 | 74.3 | 52.2 | 23.0 | 41.0 | |
| | | | 2 | 25.7 | 47.8 | 60.7 | | |
| | Sample3 | 1.296 | 1 | 72.1 | 49.5 | 22.3 | 40.8 | |
| | | | 2 | 27.9 | 50.5 | 59.0 | | |
| | | | | | | | | |
| TTA-UC@PVAc film<br><br>In Air | Sample1 | 1.160 | 1 | 34.3 | 16.4 | 37.7 | 89.8 | - |
| | | | 2 | 65.7 | 83.6 | 100 | | |
| TTA-UC@PVAc film<br><br>In $N_2$ | Sample1 | 1.216 | 1 | 21.3 | 13.4 | 60.5 | 103.6 | - |
| | | | 2 | 78.7 | 86.6 | 110 | | |

[a] $\chi$-squared, a measure of goodness of fit. [b] Normalised pre-exponential factor for $i^{th}$ component. [c] Fractional contribution of the $i^{th}$ component. [d] Lifetime of the $i^{th}$ component.

# 7 Kinetic studies on converted NBD@PVAc (QC@PVAc) film

To determine the thermal half-life of the QC@PVAc film at room temperature, UV-Vis absorbance (at 398 nm) spectra at 25, 35 and 45 °C were measured (Figure S8a-c). The absorbance changes against time were used to fit the first-order rate parameter $k$ at each temperature (as described in Eq. S6), which were used in the Eyring plot (Figure S8d). The thermodynamic parameters were calculated using the Eyring equation (Eq. S9) and thermal half-life at 25 °C calculated by extrapolating back the straight line.

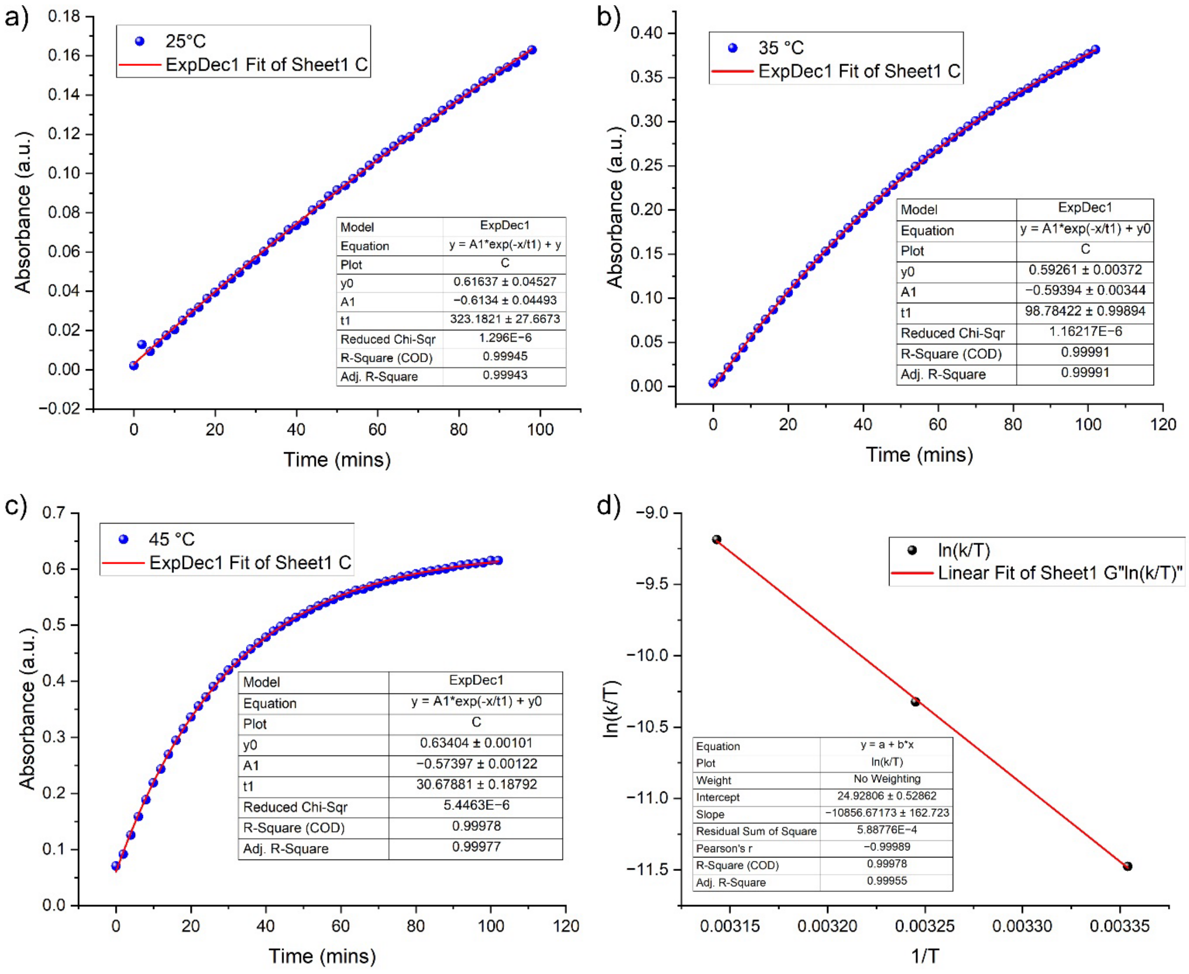


**Figure S8.** Thermal back-conversion kinetics of NBD film. (a) Thermal back-conversion kinetics at 25 °C. (b) Thermal back-conversion kinetics at 35 °C. (c) Thermal back-conversion kinetics at 45 °C. (d) Eyring plot of the NBD@PVAc film.

# 8 TTA-UC assisted photoswitching mechanism

When PtOEP molecules are excited by the irradiation light source, they undergo ISC and populate their lowest triplet excited state.

$$^{1}PtOEP + h\nu_{532\,nm} \rightarrow {}^{3}PtOEP^{*} \qquad \text{Eq. S13}$$

Then the triplet excited state of the PtOEP molecules will collide with the surrounding DPA molecules, transferring their triplet energy to populate the triplet excited state of the DPA molecules:

$$^{3}PtOEP^{*} + {}^{1}DPA \rightarrow {}^{1}PtOEP + {}^{3}DPA^{*} \qquad \text{Eq. S14}$$

Following this energy transfer process, two DPA molecules in the triplet excited states will collide again and, through triplet-triplet annihilation, emit one high-energy photon. This photon is then absorbed and converts the NBD molecule into its corresponding QC form:

$$^{3}DPA^{*} + {}^{3}DPA^{*} \rightarrow + {}^{1}DPA + {}^{1}DPA^{*} \qquad \text{Eq. S15}$$

$$^{1}DPA^{*} \rightarrow + {}^{1}DPA + h\nu_{440\,nm} \qquad \text{Eq. S16}$$

$$h\nu_{440\,nm} + NBD \rightarrow QC \qquad \text{Eq. S17}$$

# 9 TTA-UC@PVAc film fatigue test

To determine the stability of the TTA-UC@PVAc film under prolonged laser excitation (532 nm, 1 W·cm$^{-2}$), a fatigue test was conducted. The TTA-UC@PVAc film was irradiated at intervals of 0 s, 74 s, 141 s, 270 s, and 336 s. Emission spectra were recorded at each interval, and the photon counts (scan slit = 1 nm) at 440 nm were plotted against irradiation time (Figure S9).

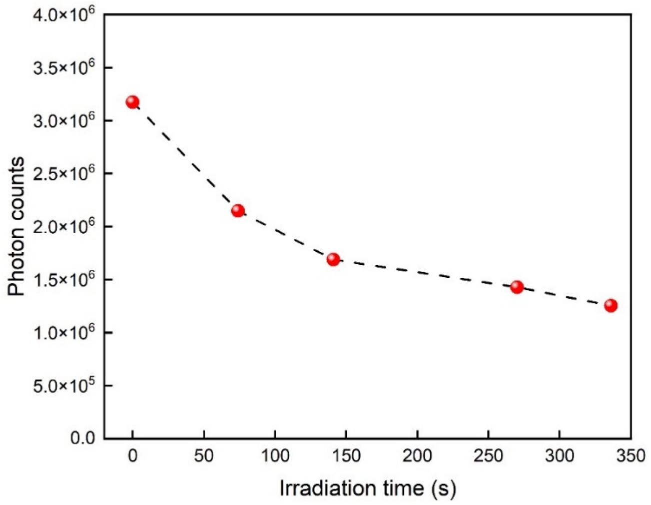


**Figure S9.** Fatigue test of the TTA-UC@PVAc film. Emission photon counts at 440 nm were recorded for the TTA-UC@PVAc film.

# 10 References

1. M. Quant, A. Lennartson, A. Dreos, M. Kuisma, P. Erhart, K. Börjesson and K. Moth-Poulsen, *Chem. Eur. J.*, 2016, **22**, 13265-13274.

2. L. Porrès, A. Holland, L.-O. Pålsson, A. P. Monkman, C. Kemp and A. Beeby, *J. Fluoresc.*, 2006, **16**, 267-273.
3. T.-S. Ahn, R. O. Al-Kaysi, A. M. Müller, K. M. Wentz and C. J. Bardeen, *Rev. Sci. Instrum.*, 2007, **78**, 086105.
4. J. C. de Mello, H. F. Wittmann and R. H. Friend, *Adv. Mater.*, 1997, **9**, 230-232.
5. J. R. Lakowicz, *Principles of fluorescence spectroscopy*, Springer, 2006.
